# Choline—An Essential Nutrient with Health Benefits and a Signaling Molecule

**DOI:** 10.3390/ijms26157159

**Published:** 2025-07-24

**Authors:** Brianne C. Burns, Jitendra D. Belani, Hailey N. Wittorf, Eugen Brailoiu, Gabriela C. Brailoiu

**Affiliations:** 1 Department of Pharmaceutical Sciences, Jefferson College of Pharmacy, Thomas Jefferson University, Philadelphia, PA 19107, USA; brianne.burns@students.jefferson.edu (B.C.B.); jitendra.belani@jefferson.edu (J.D.B.); hailey.wittorf@jefferson.edu (H.N.W.); 2 Department of Neural Sciences and Center for Substance Abuse Research, Lewis Katz School of Medicine at Temple University, Philadelphia, PA 19140, USA

**Keywords:** acetylcholine, choline transporters, G protein-coupled receptor, GPCR, phosphatidylcholine, second messenger, Sigma-1R

## Abstract

Choline has been recognized as an essential nutrient involved in various physiological functions critical to human health. Adequate daily intake of choline has been established by the US National Academy of Medicine in 1998, considering choline requirements for different ages, sex differences and physiological states (e.g., pregnancy). By serving as a precursor for acetylcholine and phospholipids, choline is important for cholinergic transmission and the structural integrity of cell membranes. In addition, choline is involved in lipid and cholesterol transport and serves as a methyl donor after oxidation to betaine. Extracellular choline is transported across the cell membrane via various transport systems (high-affinity and low-affinity choline transporters) with distinct features and roles. An adequate dietary intake of choline during pregnancy supports proper fetal development, and throughout life supports brain, liver, and muscle functions, while choline deficiency is linked to disease states like fatty liver. Choline has important roles in neurodevelopment, cognition, liver function, lipid metabolism, and cardiovascular health. While its signaling role has been considered mostly indirect via acetylcholine and phosphatidylcholine which are synthesized from choline, emerging evidence supports a role for choline as an intracellular messenger acting on Sigma-1R, a non-opioid intracellular receptor. These new findings expand the cell signaling repertoire and increase the current understanding of the role of choline while warranting more research to uncover the molecular mechanisms and significance in the context of GPCR signaling, the relevance for physiology and disease states.

## 1. Introduction

**Figure 1:**
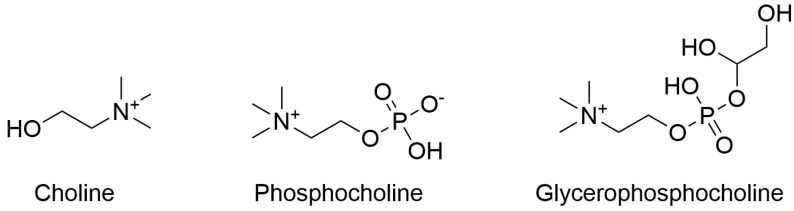
Chemical structures of choline and its related metabolites. The molecular structures of choline, phosphocholine, and glycerophosphocholine are illustrated. Phosphocholine and glycerophosphocholine are key compounds involved in choline metabolism.

Choline is a dietary component that has been recognized by the National Academy of Medicine (former Institute of Medicine) as an essential nutrient since 1998 [1]. Structurally, choline (C_5_H_14_NO^+^) is a quaternary ammonium compound characterized by a positively charged nitrogen atom bonded to three methyl groups and an ethanol group (Figure 1). Choline and its metabolites play a vital role in the body, from supporting brain function and neurotransmitter biosynthesis to maintaining liver health. Choline serves as a precursor for acetylcholine, a key neurotransmitter, important for functions such as memory, muscle control, and mood regulation [2,3]. Additionally, choline is integral to the synthesis of phosphatidylcholine and sphingomyelin, which are important for maintaining cell membrane structure and function. Choline supports lipid transport and metabolism within the liver; it is involved in the transport of lipids to various tissues by aiding in very-low-density lipoprotein (VLDL) formation, which helps prevent the accumulation of fats in the liver [4]. Furthermore, choline supports methyl group metabolism, serving as a precursor for S-adenosylmethionine, a critical methyl donor in the body. This methylation process helps maintain normal homocysteine levels, required for cardiovascular health [5]. Endogenous de novo synthesis via the sequential methylation of phosphatidylethanolamine produces limited quantities of choline, insufficient to meet physiological needs, therefore making dietary intake essential [2]. Deficiency in choline can lead to poor health outcomes, including cognitive deficit in infants [6] and liver dysfunction and muscle damage in adults [2].

## 2. Choline Intake Recommendations and Dietary Sources

Adequate daily intake (AI) of choline has been established by the US National Academy of Medicine-Food and Nutrition Board in 1998, considering choline requirements for different ages, sex differences, and physiological state, and prevention of liver damage in adults [1,7]. The choline AI increases with age: from 125 mg/day for infants (0–6 months) to 150 mg/day (6–12 months), 200 mg/day in children (1–3 years), and 250 mg/day (4–8 years). Boys and girls (9–13 years) have the same choline AI of 375 mg/day, while during adolescence (14–18 years), boys have a higher AI of choline (550 mg/day) as compared with girls (400 mg/day). A higher choline AI is needed for men (550 mg/day) as compared to women (425 mg/day) throughout the entire adult life (>19 years). The demand for choline is particularly high during pregnancy (450 mg/day) and lactation (500 mg/day), as it is critical for fetal development, particularly in brain and memory development [1,7].

**Figure 2:**
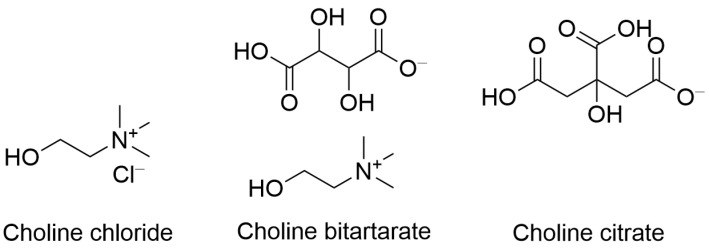
Structures of choline salts. Shown are the structures of three choline salts commonly used in dietary supplements and clinical formulations: choline chloride, choline bitartrate, and choline citrate. These salts differ in their counterions, affecting their solubility, stability, and bioavailability.

Choline and its esters are widely present in various foods, with animal products: eggs, chicken, fish, beef, and dairy products serving as particularly rich sources [7,8]. Cruciferous vegetables and soy beans are also a good source of choline, providing up to 10% of the daily requirement per serving [8]. Choline is often taken as a supplement in the form of various salts such as choline chloride, choline bitartrate, and choline citrate (Figure 2). Some multivitamin supplements, including prenatal vitamins, contain small quantities (25–50 mg) of choline [7].

In the diet, choline is present in both the water-soluble form (phosphocholine, glycerophosphocholine, free choline) and the lipid-soluble form (phosphatidylcholine and sphingomyelin) that reach the liver via portal and lymphatic circulation, respectively [9]. While the dietary choline intake varies, overall suboptimal intake of choline in the United States was determined, and only 11% of the adult population achieves the AI for choline [10]. Early evidence indicates that the plasma level of choline is maintained, relatively constant, at 10 µM [11,12]. More recently, plasma choline level evaluated by liquid chromatography-tandem mass spectrometry was identified at 15.9 μM [13]. However, the plasma choline level decreases by 50% in choline-deficient diets or can increase to 20 µM after ingestion of choline-rich foods [12]. Given the wide-ranging physiological roles of choline and the increased demand during specific life stages, ensuring sufficient dietary intake of choline is essential for long-term health and disease prevention.

## 3. Mechanisms of Transport of Extracellular Choline

The transport of choline across the cellular membranes involves different transport systems, each playing distinct roles [12,14]. The high-affinity choline transporter (CHT1) (K_m_ < 10 µM), predominantly located in cholinergic neurons, facilitates the transport of choline into presynaptic terminals, which is essential for acetylcholine production [14,15]. The choline uptake via CHT1 is sodium-dependent, inhibited by hemicholinium-3, and represents a rate-limiting step in acetylcholine synthesis. CHT1 is found in intracellular vesicles, such as endosomes and synaptic vesicles, and its presence at the plasma membrane is dynamically regulated through mechanisms involving endocytosis and exocytosis [16]. CHT1 availability at the cell surface is modulated based on neuronal activity levels with increased exocytosis of CHT1 to the plasma membrane during periods of intense cholinergic activity to meet the increased choline requirements [16], highlighting the significance of CHT1 in maintaining adequate choline levels in cholinergic neurons [12]. Conversely, dysregulation in choline transport through CHT1 is associated with neurological and psychiatric disorders such as Alzheimer’s disease, Parkinson’s disease, schizophrenia, attention-deficit hyperactive disorder (ADHD), and depression [17].

The choline transporter-like proteins family consists of five members (CTL1-5, encoded by *SLC44A1-5*) that mediate choline uptake for phospholipid synthesis in various tissues such as muscle, astrocytes, and cerebral cortex neurons [14]. The choline uptake activity of CTL1 has distinct features from CHT1 regarding the affinity for choline (intermediate versus high), sodium dependence (independent versus dependent), and inhibition by hemicholinium-3 (high Ki versus low Ki) [14].

In contrast, low-affinity choline transporters (K_m_ > 30–100 µM), such as the organic cation transporter (OCT) family, are found ubiquitously; they enable the uptake of choline for synthesis of phosphatidylcholine and other phospholipids, which is vital for cell membrane integrity [12,14]. Unlike high-affinity transporters, low-affinity systems are sodium-independent [12]. In addition to the high-affinity and low-affinity choline transporters, unique choline transport mechanisms were identified in some tissues. The blood–brain barrier (BBB) employs specific carrier-mediated and saturable mechanisms to regulate choline passage into the brain, with mixed characteristics of both low and high-affinity transporters–sodium-independent and low Km [12]. Recently, human feline leukemia virus subgroup C receptor-related proteins 1 and 2 (FLVCR1 and FLVCR2) were identified as choline transporters [18], with FLVCR2 (also known as MFSD7C and SLC49A2) being considered a BBB choline transporter, responsible for the majority of choline uptake into the brain [19,20,21]. FLVCR2 is expressed in endothelial cells of the BBB and displays characteristics of choline uniporter or proton/choline co-transporter with high affinity for choline [19]. Overall, the transporters ensure a sufficient supply of choline for both acetylcholine production in neurons and phospholipid synthesis in various tissues, adapting to the dynamic needs of the body.

## 4. Biological Roles of Choline and Implications in Disease States

### 4.1. Choline and Fetal Development

During pregnancy, choline supports the neurodevelopment and overall health of the fetus [6,22]. Adequate choline intake during pregnancy positively influences infant brain function and early cognitive development [23] and plays a protective role in preventing neural tube defects [3]. A long-term (7 years) follow-up study found that children born to mothers who took choline supplements during pregnancy exhibited improved sustained attention, suggesting that prenatal choline intake may contribute to a lower risk of attention-related disorders [24]. Choline intake during pregnancy supports brain health by maintaining membrane integrity and reducing inflammation, which may protect against neurodegenerative conditions such as Alzheimer’s disease in the long term [25]. Prenatal choline supplementation can reduce the risk of brain-related developmental disorders in offspring, suggesting its preventive value against cognitive decline and supporting brain plasticity [25]. Insufficient choline intake among childbearing-age women may compromise fetal neurodevelopment and lead to adverse cognitive outcomes [26]. These studies highlight that prenatal choline supplementation not only supports immediate fetal development but also provides long-lasting cognitive advantages in children [6,24].

Beyond brain development, choline significantly impacts fetal liver function and metabolic health. Choline has an essential role in fetal liver maturation; choline deficiency during pregnancy can result in compromised liver function, potentially predisposing the offspring to metabolic disorders later in life [27]. Choline’s support of lipid transport and cellular membrane structure underscores its foundational role in liver development, emphasizing that an adequate supply during pregnancy is vital to reduce the risk of metabolic health issues in the offspring. Given these implications, the integration of choline into prenatal dietary recommendations is essential to protect against liver dysfunction and support long-term metabolic stability [27].

The benefits of choline in pregnancy are not isolated; they often involve complex interactions with other essential nutrients, notably docosahexaenoic acid (DHA) [28,29]. Prenatal choline supplementation in women already consuming DHA improved maternal biomarkers of DHA status, enhancing DHA levels in the blood [28,29]. This elevation in DHA has been associated with favorable neurodevelopmental outcomes, suggesting that choline and DHA together provide a synergistic benefit, amplifying neurodevelopmental support for the fetus [28,29]. Lysophosphatidylcholine facilitates the brain uptake of DHA [30]. Thereby, these findings underline the importance of not only choline but also its combination with DHA in prenatal supplementation, positioning both nutrients as essential components in maternal nutrition for optimal fetal development [6,28].

Moreover, the role of choline as a methyl donor in epigenetic processes is increasingly recognized for its impact on gene expression and neurodevelopmental outcomes; choline supports DNA and histone methylation, influencing genes that are critical for neurogenesis and synaptic plasticity [31]. Through these epigenetic mechanisms, choline supplementation during pregnancy may promote neural resilience and cognitive functions such as memory and learning [31]. In addition, choline modulates the expression of SOX4, a transcription factor crucial for cortical development, through specific epigenetic pathways [32]. This epigenetic influence of choline supports that adequate intake during pregnancy could confer long-lasting neuroprotective benefits, reducing the risk of neurodevelopmental disorders and age-related cognitive decline [31,32].

### 4.2. Choline and Liver Function

Several studies in various species and humans indicate that choline is essential for normal liver function by multiple mechanisms: phospholipids derived from choline are critical components of hepatic cell membrane and contribute to lipoprotein-mediated transport of triglycerides, formation of very-low-density lipoprotein (VLDL) and secretion of triglycerides from the liver [27]. In addition, choline serves as a methyl donor after oxidation to betaine that provides S-adenosylmethionine, the cofactor for methyltransferases [27]. Choline deficiency can lead to increased oxidative stress, inflammation, and fat accumulation in the liver, which, if untreated, may progress to more severe conditions such as non-alcoholic fatty liver disease (NAFLD), cirrhosis, and liver carcinoma [4]. Choline supplementation has been proposed as a therapeutic strategy for preventing or managing NAFLD by improving lipid metabolism, reducing inflammation, and protecting liver health, particularly in individuals with genetic predispositions to impaired choline metabolism [4].

### 4.3. Choline and Cardiovascular Health

Animal studies in rodents indicate that choline has cardiovascular protective effects in arrhythmias [33,34], reduces cardiac hypertrophy [35,36], attenuates cardiac fibrosis [37] and hypertension [38] by various mechanisms. Choline reduces cardiac hypertrophy by restoring the muscle-specific microRNA miR-133a expression, an anti-hypertrophic factor, and reducing the calcineurin protein level [36]. In spontaneous hypertensive rats, choline improves cardiac function and attenuates hypertension by increasing the vagal activity and exerting anti-inflammatory effect [38]. Recent population-based studies of 14,289 participants (mean age 48.08 years) [5] and 7341 older adults (mean age 73.39 years) [39] from the National Health and Nutrition Examination Survey (NHANES) indicate that a proper dietary choline intake is correlated with a reduced risk of cardiovascular disease. However, excessive dietary choline is metabolized by intestinal microbiota to trimethylamine, oxidized to trimethylamine N-oxide (TMAO); increased TMAO levels have been involved in atherosclerosis and are associated with a higher risk of major adverse cardiovascular events [40]. Moderate choline consumption is also linked to lower all-cause mortality, suggesting a potential role for choline in promoting longevity and supporting the inclusion of choline in dietary guidelines for heart health, disease prevention, and overall longevity [5].

### 4.4. Choline in Alzheimer’s Disease and Cognitive Decline

Choline has neuroprotective potential in mitigating age-related cognitive decline, particularly in conditions like Alzheimer’s disease [41,42]. Cholinergic deficit is one of the contributing factors to the pathogenesis of Alzheimer’s disease [43]. Anticholinergic drugs may exacerbate Alzheimer’s symptoms and accelerate the cognitive decline by increasing amyloid-beta levels and reducing phosphatidylcholine [41]. Maintaining adequate choline intake could help protect against cognitive deterioration by supporting acetylcholine synthesis and neuronal health. In the APP/PS1 mouse model of Alzheimer’s disease, lifelong choline supplementation reduced the amyloid-β plaque, microglia activation, and improved the spatial memory deficits [44]. A transgenerational reduction in Alzheimer’s disease pathology was found in APP/PS1 mice offspring from a mother with a choline-enriched diet and linked to the reduction in brain homocysteine level [45]. In the Ts65Dn mouse model of Down syndrome, an increase in choline intake during gestation and lactation improved cognition of the offspring [46,47]. Choline supplementation improved cognitive performance in patients with transient global amnesia [48] and reduced the chemotherapy-induced cognitive deficit in animal models [49]. Choline administered in combination with uridine and DHA enhances synapse formation and improves cognitive function in aging population [42]. Choline and uridine work in synergy with DHA to increase the synthesis of phosphatidylcholine and improve long-term outcomes in randomized controlled trials in patients with various forms of dementia, ranging from mild cognitive impairment to moderate Alzheimer’s disease [50]. Together, these findings suggest that choline, alongside other critical nutrients, could contribute to sustaining cognitive health, reducing Alzheimer’s disease pathology, and promoting brain resilience [41,42].

### 4.5. Choline and Addiction

Choline has shown promising potential as a therapeutic intervention for children with Fetal Alcohol Spectrum Disorder (FASD), a condition associated with prenatal alcohol exposure leading to cognitive deficits [51]. Choline intake improved cognitive performance, particularly in children diagnosed with FASD who have specific genetic variations in the *SLC44A1* gene, which is involved in choline transport. These children exhibited greater cognitive gains in response to choline, suggesting that genetic factors may influence the effectiveness of choline as a therapeutic intervention, and raising the possibility of personalized choline supplementation for children affected by FASD. Overall, these findings highlight the importance of adequate choline intake in managing neurodevelopmental disorders like FASD and support further exploration of choline’s role in mitigating cognitive impairments linked to prenatal alcohol exposure [51]. We recently reported that choline is involved in the potentiation of orexin A signaling by cocaine, a drug of abuse [52]. Orexin A is an endogenous peptide involved in regulating wakefulness, energy metabolism, and reward [53,54].

### 4.6. Choline and Cancer

**Figure 3:**
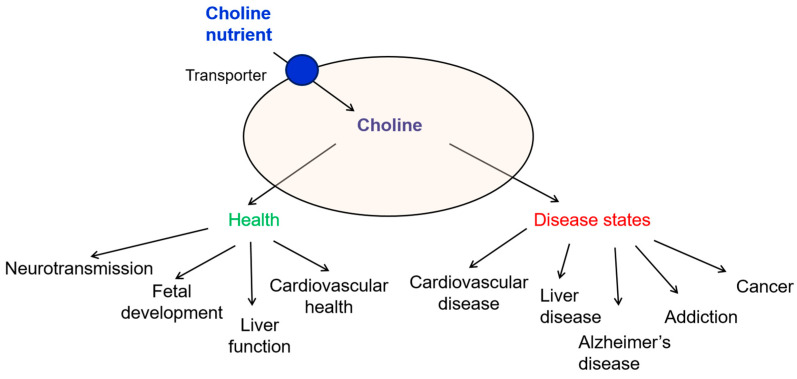
Diagram summarizing the roles of choline in health and disease states. Choline, an essential nutrient, can be transported intracellularly via various transport mechanisms. It serves as a precursor for acetylcholine, a critical neurotransmitter, and contributes to fetal development, liver function, and cardiovascular health. Choline deficit has been involved in cardiovascular diseases, liver dysfunction, Alzheimer’s disease, and addiction, while both deficit and excess of choline were associated with cancer.

The relationship between choline intake and the risk of cancer remains a complex area of study. Choline deficiency can lead to liver dysfunction that may progress to fibrosis, cirrhosis, and liver cancer [4,55]. Population-based case–control studies and a meta-analysis of epidemiologic studies [56] indicate that high intake of choline and betaine reduced the risk of breast cancer [57], esophageal cancer [58], lung cancer [59], nasopharyngeal cancer [60], colon cancer [5], while did not impact the risk of renal cancer [61] or ovarian cancer [62]. A recent systematic review of choline metabolism in oncology [63] highlights two additional points. First, existing literature still focuses primarily on dietary intake rather than plasma choline levels. Second, in the limited studies that do measure circulating choline, plasma concentrations are mostly inversely associated with incident colorectal and pancreatic cancer, although one nested case–control analysis reported a positive colorectal signal. Moreover, studies measuring plasma choline levels were small and subject to selection bias [63]. Other studies found an increase in the risk of prostate cancer [64,65,66] or colorectal cancer [67] with higher choline intake. These findings suggest that while there may be a weak link between choline and cancer risk, it is not strong enough to warrant dietary changes solely for cancer prevention. Larger, prospective studies that integrate both dietary and plasma choline measures across diverse cancer types are needed to clarify any relationship between choline level and cancer risk and to better understand the impact of dietary interventions across different cancer types [5,63,64,67]. A summary of various roles of choline in physiology and disease states is provided in Figure 3.

## 5. Detection of Choline in Biological Samples

Accurate detection and quantification of choline in plasma and tissue samples are essential to fully understand its roles in metabolism, signaling, and disease pathology. Nuclear magnetic resonance spectroscopy (NMR), mass spectrometry (MS), and high-performance liquid chromatography (HPLC) have all been used to identify and quantify choline and related compounds. One of the original methods of detection used HPLC separation followed by electrochemical detection of hydrogen peroxide released from the reaction of choline with choline oxidase [68]. The method was subsequently used to measure choline in blood plasma with a linear response in the 1–20 μmol/L range [69]. Improvements in high-throughput detection methods, such as LC-MS and LC-MS/MS, have significantly enhanced the sensitivity, specificity, and efficiency of choline analyses [13]. Moreover, isotope dilution [70], enhanced sample preparation methods [71], and optimized chromatographic conditions [72] have helped improve the accuracy of choline quantification. The detection limits have also improved with studies demonstrating detection as low as 5 ng/L of choline, with strong accuracy and precision [13,73]. Although LC-MS remains significantly more sensitive to the detection of choline and its derivatives [73,74], NMR has played an alternate and important role in their detection and quantification. Initially, high-field ^1^H and ^31^P-NMR spectroscopy was used to quantify total choline as a cancer biomarker and it enabled differentiation between phosphocholine and glycerophosphocholine in tumor tissues [75]. Quadrupolar ^14^N NMR has been explored as an alternative detection method, leveraging the higher natural abundance and sensitivity of ^14^N, despite challenges related to probe compatibility [76]. More recently, advances in NMR hardware and analytical algorithms have improved the clinical and regulatory relevance of NMR methods. A clinical NMR-based assay was developed to quantify choline with good sensitivity and reproducibility using the Vantera^®^ clinical analyzer [77]. This method employs a non-negative deconvolution algorithm to isolate choline’s spectral signal and demonstrates a strong correlation with LC-MS/MS (R = 0.998), with quantification limits of 7.1 μmol/L in serum and 5.9 μmol/L in plasma [77]. In a separate study reporting threshold impurity for pharmaceutical quality control meeting International Council for Harmonisation (ICH) requirements for impurity detection, NMR has demonstrated the ability to detect choline impurities, such as O-(2-hydroxyethyl)choline, at levels as low as 0.01% in choline chloride samples using high-field ^1^H NMR spectroscopy [78]. These results reject the widespread assumption that NMR lacks sufficient sensitivity for impurity analysis and highlights its utility even at benchtop field strengths under optimized conditions. Furthermore, de Graaf et al. recently described a 2D-^1^H–^14^N heteronuclear single-quantum coherence (HSQC) NMR method that enables simultaneous detection of both protonated and deuterated choline metabolites, including choline, phosphocholine, glycerophosphocholine, CDP-choline, and betaine, in excised tissues [79]. The technique improves analytical resolution and facilitates metabolic tracing of exogenous choline sources by utilizing scalar coupling between ^14^N and CH_2_ protons and the chemical shift sensitivity of ^14^N to nearby deuterium, enabling high-resolution discrimination of metabolite species. This capability is especially important in experimental and nutritional studies, and it enhances the analytical resolution beyond traditional ^1^H or ^2^H magnetic resonance spectroscopy. It also supports metabolic tracing using deuterated choline (e.g., D_9_-choline) and enables quantification of isotopic enrichment alongside absolute concentration measurements [79]. These advances have transformed choline detection from general quantification to precise molecular profiling, allowing insights into dynamic metabolism, impurity control, and metabolic imaging. Once viewed as less sensitive than MS, NMR now offers strong, complementary capabilities that are especially valuable in metabolic tracing, clinical diagnostics, and pharmaceutical quality assurance.

## 6. Second Messenger Role for Choline Acting on Sigma-1R

**Figure 4:**
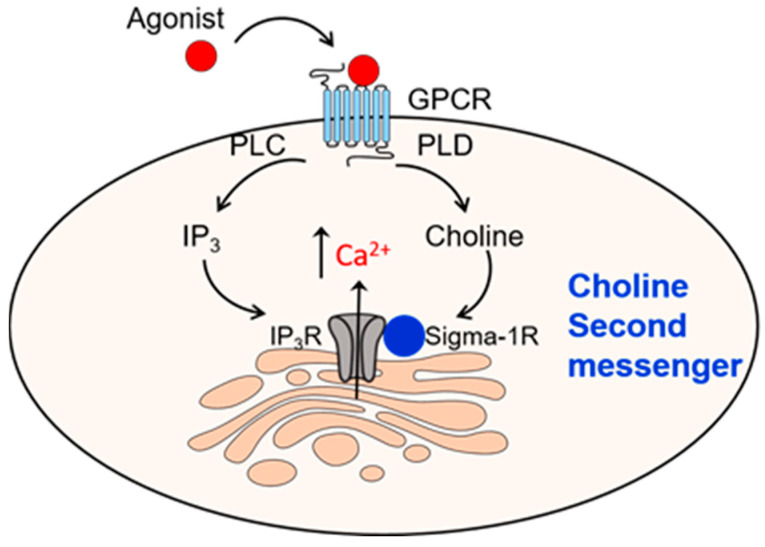
Diagram illustrating the second messenger role of choline. G protein-coupled receptor (GPCR) agonists that stimulate phospholipase C (PLC) and phospholipase D (PLD) lead to consequent formation of inositol 1,4,5-trisphosphate (IP_3_) and choline. IP_3_ stimulates IP_3_ receptor (IP_3_R), while choline binds to Sigma-1 receptors (Sigma-1R), and potentiates IP_3_R activity.

The signaling role of choline has been considered mostly indirect via acetylcholine and phosphatidylcholine synthesized from choline. Phospholipase D (PLD) hydrolyzes phosphatidylcholine, the most abundant membrane phospholipid in mammalian cells, releasing choline and phosphatidic acid (PA) [80,81]. PA has been considered the main signaling molecule produced from phosphatidylcholine [80]. Relatively recently, we have identified that choline acts as an intracellular messenger that links extracellular stimuli to intracellular calcium signaling pathways by activating Sigma-1 receptors (Sigma-1R) [82], a non-opioid intracellular receptor located on the endoplasmic reticulum [83,84,85,86,87,88]. Sigma-1Rs bind various ligands, most of which are amines, such as antidepressants (e.g., fluoxetine), antipsychotics (e.g., haloperidol), and drugs of abuse (e.g., cocaine and methamphetamine) [88,89,90,91,92]. Choline, but not its metabolites phosphocholine or betaine, binds Sigma-1R and enhances inositol 1,4,5-trisphosphate (IP3)-evoked Ca^2+^ release from the endoplasmic reticulum [82]. Therefore, G-protein coupled receptors (GPCRs) signal to IP_3_Rs through two pathways, IP_3_ and choline, that converge to the stimulation of IP_3_Rs (Figure 4). IP_3_ is generated together with diacylglycerol (DAG) from phosphatidylinositol-4,5-bis-phosphate (PIP2) by phospholipase C (PLC). Several GPCR agonists such as bradykinin, angiotensin II, endothelin-1, carbachol, orexin, and thyroid-stimulating hormone activate PLD and PLC [93,94,95,96,97]. Gq-coupled receptors like AT1 receptor or muscarinic M3 receptor activate PLC and PLD via RhoA and PKC-dependent process [96,98], while other GPCRs, like alpha-1 adrenergic receptors activate PLD via a PKC-independent process [97,99].

The basal PLD activity in mammalian cells is low and transiently increases in response to receptor activation [94]. Mammals have six different PLD enzymes, with PLD1 and PLD2 being the best characterized; there are 2 splice variants for PLD1 and 3 splice variants for PLD2 [100]. PLD isoenzymes have a wide tissue distribution; at the cellular level, PLD1 is localized in the endoplasmic reticulum, Golgi and endosomes [101]; PLD2 is present in the plasma membrane [102]; PLD3 and PLD4 are localized to lysosomes [103,104] and PLD5 and PLD6 are localized to mitochondria [104].

Choline meets the five criteria for a second messenger formulated by Sutherland [105,106,107]. The first criterion set out by Sutherland for a second messenger is that antagonism of the action of the messenger blocks the effects of the extracellular messenger. In support of this criterion, we found that the Ca^2+^ signals evoked by bradykinin, a GPCR agonist that stimulates PLC and IP_3_R-evoked increase in Ca^2+^, were attenuated by BD 1047, a Sigma-1R antagonist [52,108] in NG108-15 cells, neuroblastoma-glioma cells that express Sigma-1R [82]. Reduction in Sigma-1R expression by transfection of cells with Sigma-1R shRNA reduced the amplitude of Ca^2+^ signals produced by bradykinin or ATP [82] another GPCR agonist that stimulates PLC via P2Y6 receptors in NG108-15 cells [109].

Sutherland’s second criterion is that when the molecule is applied intracellularly, it must mimic the effect of an extracellular stimulus. Multiple lines of evidence indicate that in different cell types Sigma-1Rs potentiate the IP_3_-evoked increase in cytosolic Ca^2+^ concentration [110,111,112]. To address this criterion, we determined the effect of microinjection of choline alone or in co-injection with IP_3_ on cytosolic Ca^2+^ concentration; microinjection of choline potentiated the IP_3_-evoked Ca^2+^ signals in cells endogenously expressing Sigma-1R or transfected with the receptor [82] similarly to the potentiation produced by other agonists [112,113].

The third criterion for the second messenger is that it can be synthesized and metabolized. The pathways for synthesis and metabolism of choline are well-characterized and widely accepted: choline is synthesized by PLD from phosphatidylcholine [80] and is metabolized by phosphorylation to phosphocholine, an inactive derivative, or by oxidation to betaine in the kidney, liver, and brain [114].

The fourth criterion of Sutherland is that the second messenger levels change in response to a physiologically relevant stimulus. Stimulation of NG108-15 cells with ATP increased intracellular choline and IP_3_ levels; knockdown of PLD1 and PLD2 using shRNA prevented the ATP-induced increase in choline, while it did not affect the IP_3_ level [82]. These results indicate that stimulation with ATP promotes choline synthesis via a PLD-dependent mechanism.

Sutherland’s fifth criterion for a second messenger is the presence of specific intracellular binding sites. To address this criterion, we performed a competitive binding assay in membranes prepared from Neuro-2A cells stably expressing Sigma-1R incubated with [3H](+) pentazocine, a high-affinity selective Sigma-1R ligand [115] and choline. Choline completely displaced the specific binding of [3H](+) pentazocine (K_i_ = 525 µM) while phosphocholine, the major choline metabolite, did not displace it; betaine and acetylcholine were less effective than choline [82]. These results support that choline binds to greater affinity than its metabolites to the same site as Sigma-1R ligands [116,117].

Sigma-1R has been considered a promising therapeutic target for several neurological conditions such as Alzheimer’s, Huntington’s and Parkinson’s disease, epilepsy, amyotrophic lateral sclerosis [118,119,120,121,122,123,124,125,126,127,128,129], cognitive and affective disorders [130], psychiatric diseases [131], neuropathic pain [132,133,134], cardiovascular diseases [135,136,137], chronic kidney disease [137,138] and cancer [139,140,141].

Choline-Sigma 1R signaling downstream to GPCR activation is an emerging new concept with potential implications for substance use disorders and eating disorders [52,142,143], spatial memory [130], cognition [44], blood–brain barrier permeability [144], cardiac fibrosis [37] and cancer [66,141]. This new signaling mechanism has been mentioned in the context of Ca^2+^ signaling in oomycetes [145], as a potential mechanism for the antiviral effect of choline in microglial cells [146] and in the endoplasmic reticulum-mitochondrial calcium handling via FLVCR1a (feline leukemia virus subgroup C receptor 1) [147]. Moreover, PLD dysregulation and choline-Sigma1R may play a role in colorectal cancer and glioblastoma via cross-talk with PI3K-Akt/Wnt/β-catenin pathways [148].

The concept of choline as a second messenger downstream to GPCR stimulation enriches the cellular signaling repertoire and supports the need for further studies to investigate the molecular mechanisms through which GPCR agonists generate choline, to understand interactions with other second messengers and to elucidate its significance in health and disease states.

## Abbreviations

The following abbreviations are used in this manuscript:

AI	Adequate daily intake
CHT1	High-affinity choline transporter
CTL	Choline transporter-like proteins
DAG	Diacyl glycerol
DHA	Docosahexaenoic acid
FASD	Fetal Alcohol Spectrum Disorder
GPCR	G protein-coupled receptor
IP_3_	Inositol 1,4,5-trisphosphate
IP_3_R	Inositol 1,4,5-trisphosphate (IP_3_) receptor
PKC	Protein kinase C
PLC	Phospholipase C
PLD	Phospholipase D
VLDL	Very-low-density lipoprotein
